# The Non-JAZ TIFY Protein TIFY8 from *Arabidopsis thaliana* Is a Transcriptional Repressor

**DOI:** 10.1371/journal.pone.0084891

**Published:** 2014-01-08

**Authors:** Amparo Cuéllar Pérez, Astrid Nagels Durand, Robin Vanden Bossche, Rebecca De Clercq, Geert Persiau, Saskia C. M. Van Wees, Corné M. J. Pieterse, Kris Gevaert, Geert De Jaeger, Alain Goossens, Laurens Pauwels

**Affiliations:** 1 Department of Plant Systems Biology, VIB, Gent, Belgium; 2 Department of Plant Biotechnology and Bioinformatics, Ghent University, Gent, Belgium; 3 Plant-Microbe Interactions, Institute of Environmental Biology, Department of Biology, Faculty of Science, Utrecht University, Utrecht, The Netherlands; 4 Department of Medical Protein Research, VIB, Gent, Belgium; 5 Department of Biochemistry, Ghent University, Gent, Belgium; Instituto de Biología Molecular y Celular de Plantas, Spain

## Abstract

Jasmonate (JA) signalling is mediated by the JASMONATE-ZIM DOMAIN (JAZ) repressor proteins, which are degraded upon JA perception to release downstream responses. The ZIM protein domain is characteristic of the larger TIFY protein family. It is currently unknown if the atypical member TIFY8 is involved in JA signalling. Here we show that the TIFY8 ZIM domain is functional and mediated interaction with PEAPOD proteins and NINJA. TIFY8 interacted with TOPLESS through NINJA and accordingly acted as a transcriptional repressor. *TIFY8* expression was inversely correlated with *JAZ* expression during development and after infection with *Pseudomonas syringae*. Nevertheless, transgenic lines with altered TIFY8 expression did not show changes in JA sensitivity. Despite the functional ZIM domain, no interaction with JAZ proteins could be found. In contrast, TIFY8 was found in protein complexes involved in regulation of dephosphorylation, deubiquitination and O-linked N-acetylglucosamine modification suggesting an important role in nuclear signal transduction.

## Introduction

Jasmonates (JAs) are plant-specific hormones that regulate processes such as vegetative growth, cell cycle progression, trichome formation, senescence, male fertility and responses to both abiotic and biotic stresses. JAs are known to control the production of a myriad of species-specific secondary metabolites. Moreover, JA signals can be integrated with signals of other plant hormones such as auxins, abscisic acid, ethylene, gibberellins and salicylic acid (SA), which fine-tunes different responses, for example during plant defence [1]–[7]. Conversely, pathogens have developed diverse mechanisms to suppress plant defences and successfully infect the plants [7], [8]. One of the best studied cases is the hemibiotrophic bacterial pathogen *Pseudomonas syringae*. Several pathovars of this species produce the phytotoxin coronatine (COR), an important virulence factor and a structural analogue of (+)-7-*iso*-Jasmonoyl-L-isoleucine (JA-Ile), the bioactive form of JAs [9]. Following *P. syringae* infection, COR mimics JA-Ile, and thereby induces the JA signalling pathway. In turn, the activation of the JA-responses partially inhibits the SA-dependent defence responses that are triggered after *P. syringae* infection, thereby allowing bacterial colonization and symptom development [7], .

The discovery of the JASMONATE-ZIM DOMAIN (JAZ) proteins signified a breakthrough in the study of JA perception and signalling [14]–[16]. JAZ proteins act as negative regulators of JA signalling. In the absence of JAs, they bind and repress multiple transcription factors controlling the expression of JA-responsive genes. The presence of JA-Ile targets JAZ proteins for proteasomal degradation, releasing the transcription factors to regulate JA-dependent gene expression [17], [18].

**Figure 1:**
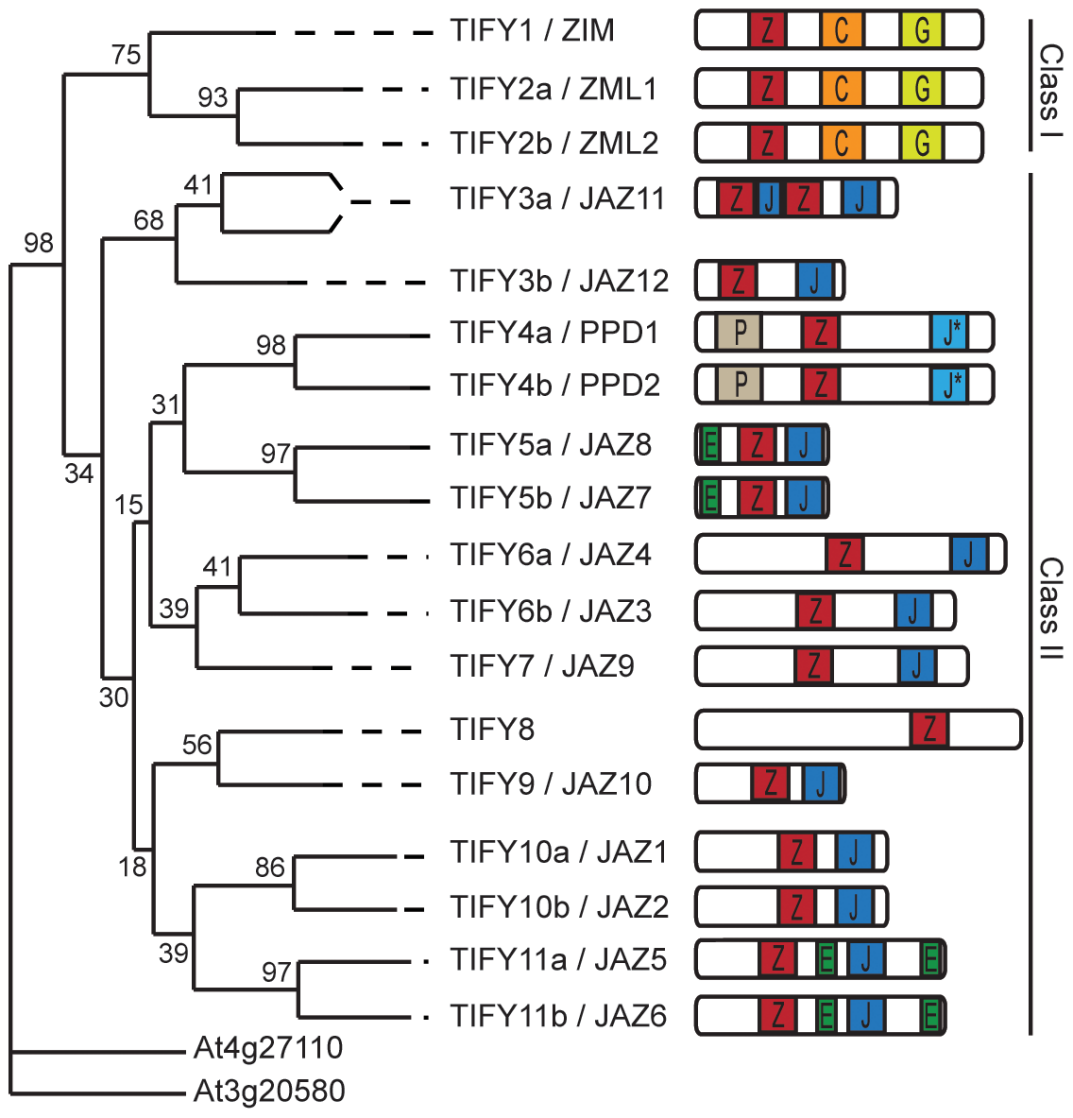
The TIFY protein family in Arabidopsis. Phylogenetic tree of the Arabidopsis TIFY family members based on the ZIM domain (Z) protein sequence. AT4G27110 and AT3G20580 were chosen as the outgroup. AT4G27110 contains a TIFY motif but is not conserved in the domain outside this motif. Consequently, it is not considered to be a real TIFY protein. The second protein, AT3G20580, is its closest homologue within the parsed region. The numbers above the branches are bootstrap values from 100 replicates and assess the robustness of the tree. Additional protein domains are shown. C: CONSTANS, CO-like, and TOC1 (CCT) domain; G: C2C2-GATA Zn-finger; P: PEAPOD domain; J: Jas domain; J* Jas-like domain; E: EAR domain. Figure adapted from [20].

Arabidopsis (*Arabidopsis thaliana*) has 12 JAZ proteins that belong to the plant-specific TIFY family, named after the core TIF[F/Y]XG motif within the Zinc-finger protein expressed in Inflorescence Meristem (ZIM) protein domain, conserved amongst all the family members [19], [20]. The TIFY family can be divided in two classes, according to the presence of a C2C2-GATA domain (Figure 1). The Arabidopsis genome harbours three proteins that contain a C2C2-GATA and a divergent ZIM domain, ZIM (At4g24470), ZIM-LIKE1 (ZML1, At3g21175), and ZML2 (At1g51600), which are classified as group I TIFY proteins. None of the 12 JAZ proteins contains the C2C2-GATA domain and thus all belong to Class II. Other members of class II are TIFY8 (At4g32570) and the PEAPOD (PPD) proteins PPD1 (At4g14713) and PPD2 (At4g14720) (Figure 1).

The different domains present in the JAZ proteins (Figure 1) provide the specificity for protein-protein interactions that determine the differential formation of complexes in the absence or presence of the hormone [18]. All 12 JAZ proteins possess a C-terminal Jas domain [16], which mediates interaction with several transcription factors, including several bHLH- and R2R3-MYB-type factors that regulate different JA-dependent responses [18]. The Jas domain also mediates the interaction of JAZ proteins with CORONATINE-INSENSITIVE1 (COI1) [14]–[16]. COI1 is the F-box subunit of SCF^COI1^, an E3-ubiquitin ligase complex [21], [22]. JA-Ile or COR can act as a “molecular glue” between COI1 and the JAZ proteins [23]–. This interaction targets the JAZ proteins for 26S-mediated proteasomal degradation.

The ZIM domain is known to mediate homo- and heterodimerization between JAZ proteins [27], [28] and to exert the repressor function of the JAZ proteins, as it enables the recruitment of the co-repressor TOPLESS (TPL) through interaction with the NOVEL INTERACTOR OF JAZ (NINJA) protein [29], [30]. NINJA possesses an ETHYLENE RESPONSE FACTOR (ERF)–associated amphiphilic repression (EAR) motif through which it can interact with TPL. A subset of the JAZ proteins, i.e. JAZ5 to JAZ8, has been found to contain EAR motifs as well [31] (Figure 1) and were recently reported to be capable of directly interacting with TPL without a need for NINJA [32]–[34].

Compared to the JAZs, the PPD1 and PPD2 proteins contain an additional N-terminal PPD-domain and a divergent C-terminal Jas domain [19]. PPD proteins have been described to regulate leaf lamina size [35] and the PPD2 protein was reported as an interactor of the coat protein promoter of the *Tomato golden mosaic virus*, suggesting DNA-binding activity [36].

The TIFY8 protein (encoded by *At4g32570*) is an atypical TIFY family member for which no other specific protein domains besides the ZIM domain have been described. TIFY8 has been reported to interact with the NINJA adaptor protein and a wide set of proteins in yeast two-hybrid assays [29], [34], and to be downregulated upon *Cabbage leaf curl virus* infection [37]. To date, its function remains unknown.

In this study, we report on the characterization of TIFY8. We confirm the ZIM domain to be functional and mediate interaction with NINJA-TPL and PPD proteins. *TIFY8* expression or TIFY8 stability were not affected by JA treatment, nor was interaction with known JA-signalling proteins besides NINJA observed. Instead, Tandem Affinity Purification (TAP) of TIFY8 identified multiple interaction partners including possible transcriptional regulators involved in plant growth and development. Accordingly, overexpression of *TIFY8* did not lead to altered JA responses but was correlated with reduced root growth.

## Results

### The TIFY8 protein is an atypical TIFY protein

A phylogenetic tree based on the ZIM domain sequence shows that the TIFY8 ZIM domain is closely related to that of the JAZ proteins, but that TIFY8 lacks additional domains present in the TIFY family such as the CCT, C2C2-GATA, EAR, Jas or PPD domains (Figure 1) [20]. This atypical domain structure prompted us to study TIFY8 conservation in the plant kingdom. According to the PLAZA comparative genomics platform (http://bioinformatics.psb.ugent.be/plaza; [38]) TIFY8 is present in the fern *Selaginella moellendorffii*, the moss *Physcomitrella patens* and in dicots, but appears to be lost in monocots. Within the dicots studied, TIFY8 orthologues are mostly present as unique genes, including in Arabidopsis (Figure S1).

### TIFY8 contains a functional ZIM domain

**Figure 2:**
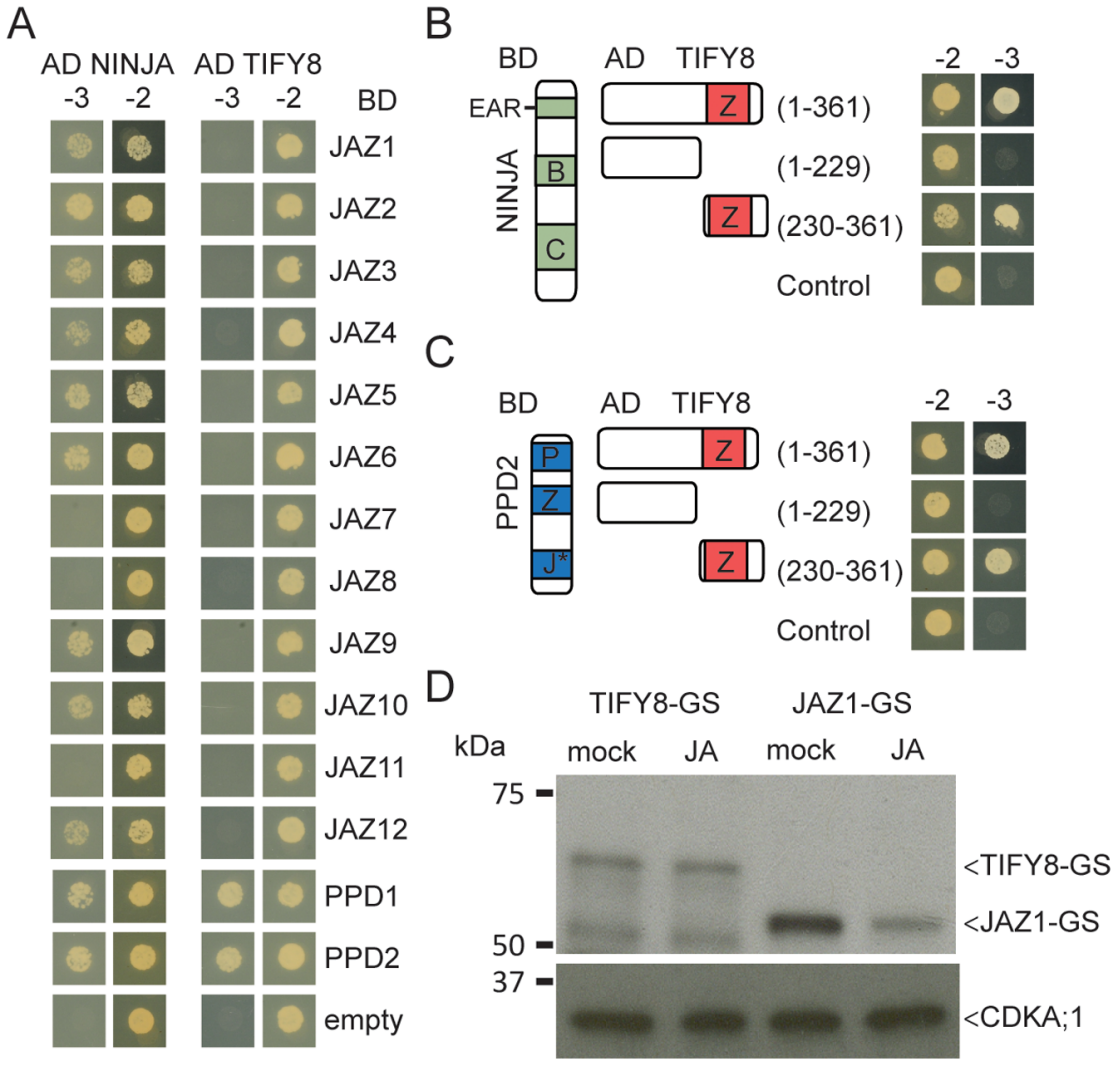
The ZIM domain of TIFY8 is functional. **A**, Y2H analysis of TIFY8 interaction with class II TIFY proteins. NINJA was included as a positive control for JAZ interaction. **B, C**, Analysis of TIFY8 truncations to map the interaction domain with NINJA (**B**) and PPD2 (**C**). Co-transformation of the PJ69-4A yeast strain with TIFY8 or NINJA and all TIFY family members in Gateway-compatible pGADT7 and pGBKT7 vectors, respectively. Transformed yeasts were spotted on control medium lacking Leu and Trp (-2) or selective medium additionally lacking His (-3). AD: activation domain; BD: DNA-binding domain. Controls for autoactivation are provided by transformation with the corresponding empty vector. **D**, Immunoblot analysis of 7 day-old Arabidopsis seedlings overexpressing the TIFY8- or JAZ1-GS fusions after 1 h treatment with either 50 µM JA or ethanol (mock). Immunoblot using the Peroxidase Anti-Peroxidase (PAP) (top) and anti-cdc2 (bottom) antibodies.

The ZIM domain is known to mediate homo- and heterodimerization of JAZ proteins [27], [28] and interaction with NINJA [29]. To investigate if the ZIM domain sequence observed in TIFY8 is functional, we tested interaction of TIFY8 with all group II TIFY proteins and NINJA. Direct binding of TIFY8 to both NINJA and PPD proteins was observed, but not to any of the JAZ proteins (Figure 2A). Interaction of all class II TIFY proteins with NINJA was tested in parallel as a control and confirmed interaction with most JAZ as previously reported [29]. TIFY8 homodimerization could not be assessed due to autoactivation. Finally, using truncations of TIFY8 we confirmed that the ZIM domain of TIFY8 is necessary and sufficient for the interaction with NINJA and PPD2 (Figure 2B–C).

The TIFY8 protein lacks the Jas-domain, typical for the JAZ proteins and required for their interaction with COI1. We tested stability of the TIFY8 protein in the presence of JAs. Arabidopsis seedling cultures producing protein G/streptavidin-binding peptide (GS)-tagged TIFY8 protein (see also below) were treated for 1 h with 50 µM JA and protein accumulation was scored by immunoblot analysis. TIFY8-GS accumulation was not affected by JA treatment, in contrast to that of GS-tagged JAZ1 proteins, which were largely degraded within 1 h of JA application (Figure 2D). Together, these data support that the TIFY8 protein is stable upon JA treatment corresponding to the absence of a Jas domain.

### Identification of TIFY8 interacting proteins

To unravel the molecular function of TIFY8, TAP in Arabidopsis cell cultures [39] was performed. GS-tagged TIFY8 (TIFY8-GS) was stably expressed under control of the CaMV 35S promoter and TIFY8 protein complexes were purified. The experiment was performed using MALDI-TOF/TOF peptide identification and on independent purifications with Orbitrap mass spectrometry, shown to increase the number of detected interactor-derived peptides [40]. In the latter experiments we also included samples of cell cultures treated for 1 h with JA.

We could confirm interaction with NINJA and PPD2. Moreover, new proteins not previously associated with NINJA or JAZ proteins were retrieved (Table 1, Table S1 and Dataset S1). Remarkably, TPL was identified only after JA treatment, a fact that might be explained by an increase in the TPL protein pool available for interaction with TIFY8 following JA-mediated degradation of the JAZ proteins. In agreement with the Y2H analysis, TAP with TIFY8 did not retrieve any of the JAZ proteins as potential interactors.

**Table 1:** Overview of prey proteins identified through TAP using TIFY8 as bait.

AGI	Protein	MALDI TOF/TOF mock	Orbitrap Mock	Orbitrap 50 µM JA
**TIFY proteins**				
AT4G32570	TIFY8	2	2	2
AT4G14720	PPD2	2	2	2
**Repressor proteins**				
AT4G28910	NINJA	2	2	2
AT1G15750	TPL			2
**Other**				
AT4G32295	Unknown	2	2	2
AT3G24150	Unknown		2	2
AT3G11540	SPY		2	2
AT1G51690	ATB alpha		2	2
AT5G48160	OBE2		2	2
AT3G63500	TTA2		2	2
AT3G08530/AT3G11130	Clathrin, heavy chain		1	2
AT4G23460/AT4G11380	Adaptin family protein		1	1
AT3G25800	PP2A-4			2
AT2G42500	PP2A-3			2
AT5G06600	UBP12			2

Proteins were identified using peptide-based homology analysis of MS data. Background proteins identified in control experiments were withdrawn. Number indicates the times the prey was identified in 2 experiments with each bait protein. Abbreviations: AGI, Arabidopsis Genome Identifier; PPD2, PEAPOD2; NINJA, NOVEL INTERACTOR OF JAZ; TPL, TOPLESS; SPY, SPINDLY; TTA2, TITANIA2; PP2A, PROTEIN PHOSPHATASE2A; UBP12, UBIQUITIN-SPECIFIC PROTEASE. Detailed MS data can be found in Table S1 and Dataset S1.

Our results reflect a wide diversity within the TIFY8 interactors (Table 1); suggesting TIFY8 may have pleiotropic roles. For instance, several protein phosphatases (PP2As) and an ubiquitin protease (UBP12) are detected. The latter has been shown to be an active ubiquitin protease that, together with its homologue UBP13, negatively regulates plant immunity [41]. Two PHD-finger proteins, OBERON2 (OBE2) and TITANIA (TTA2), are also found. These two proteins belong to a small protein family formed by 4 members (OBE1/2 and TTA1/2), which play a role in regulating MONOPTEROS-mediated gene expression during embryonic root meristem initiation [42], [43]. Also the N-acetylglucosaminyltransferase SPINDLY (SPY) was retrieved. SPY is known to function as a negative regulator of GA signalling and also mediates cytokinin responses in leaves and flowers [44], [45]. Finally two homologous proteins of unknown function were retrieved, encoded by At4g32295 and At3g24150.

### TIFY8 recruits TOPLESS via NINJA

**Figure 3:**
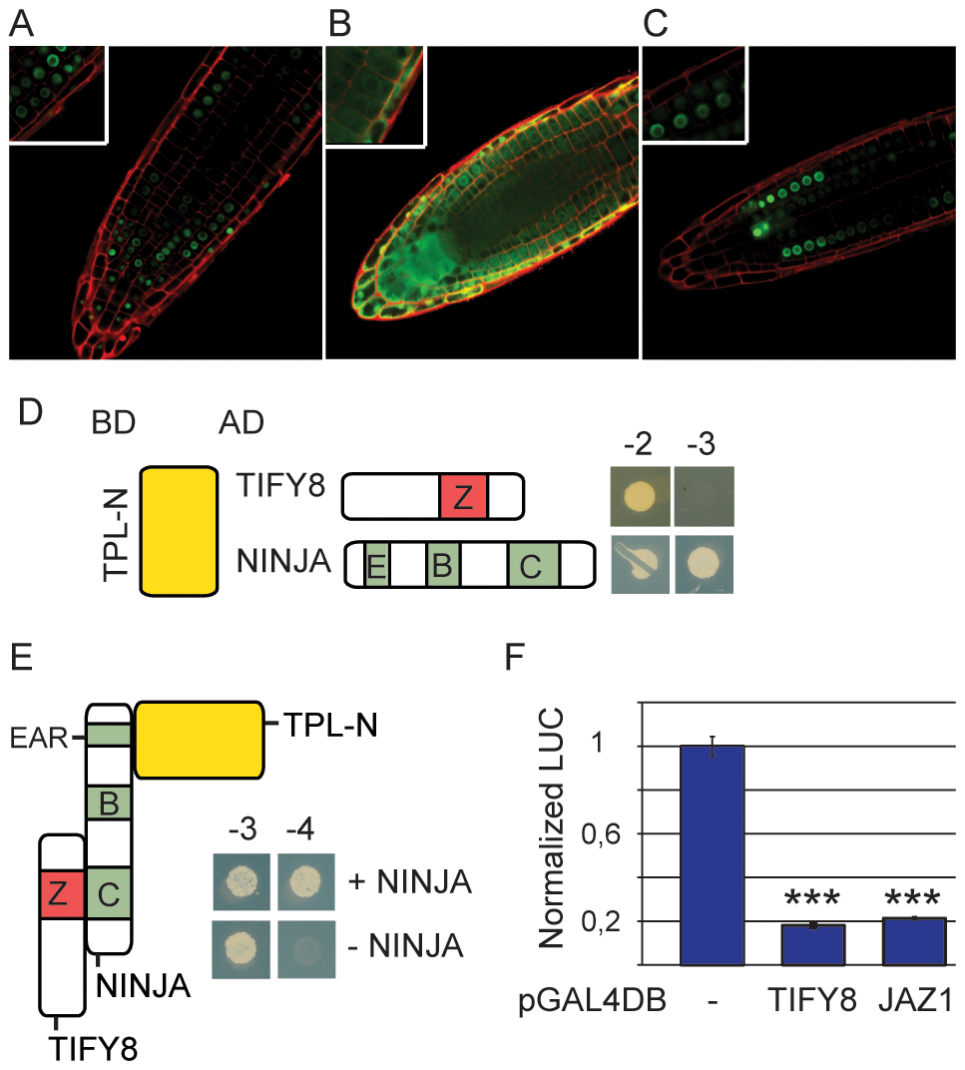
TIFY8 is a nuclear transcriptional repressor. **A**–**C**, TIFY8 localizes to the nucleus. Confocal root tip imaging of 4-day-old Arabidopsis seedlings overexpressing the TIFY8-GFP fusion protein (**A**), free GFP (**B**) or SV40-NLS fused GFP (**C**), respectively. Propidium iodide staining was performed prior to imaging to enhance the visualization of the cells. **D**, NINJA, but not TIFY8, interacts directly with TPL in Y2H assays. Co-transformation of the PJ69-4A yeast strain with TIFY8 or NINJA and the N-terminal fragment of TPL (TPL-N) in pGADT7 or pGBKT7 vectors, respectively. Transformed yeast were spotted on control medium lacking Leu and Trp (-2) or selective medium additionally lacking His (-3). **E**, TIFY8 recruits TPL through interaction with NINJA in Y3H assays. Co-transformation of the PJ69-4A yeast strain with TIFY8 and TPL-N in Gateway-compatible pGADT7 and pGBKT7 vectors, respectively, together with NLS-3xFLAG-6xHis tagged NINJA in the pMG426 vector. Transformed yeast were spotted on control medium lacking Leu, Trp and Ura (-3) or selective medium additionally lacking His (-4). A negative control is provided by substitution of NINJA by the empty pMG426 vector. **F**, TIFY8 acts as a transcriptional repressor in transient expression assays. Transactivation activity in tobacco protoplasts transfected with a pUAS–fLUC reporter construct, effector constructs fused to GAL4DBD, and a 35S:rLUC normalization construct. Error bars represent ±SE of eight biological replicates. Asterisks represent significant differences (***, p<0.001, one-way ANOVA, Tukey HSD's Post Hoc test).

We further studied the interaction with NINJA and TPL, given our interest in these proteins and their established role in orchestrating repression of gene expression [29]. First, we assessed the intracellular localization of TIFY8. Confocal imaging of Arabidopsis plants expressing a TIFY8-GFP fusion protein showed that TIFY8 localizes to the nucleus (Figure 3A–C). This also correlates with the proven nuclear localization of many of the interacting proteins detected by TAP, such as NINJA, PPD, SPY or OBE2 [29], [36], [46], [47]. JAZ proteins function as transcriptional repressors by recruiting the repressor protein TPL through NINJA, forming a ternary repression complex [29]. Hence, we studied the capacity of TIFY8 to form such repressor protein complexes. Since TIFY8 lacks an EAR motif itself, it likely cannot directly interact with TPL. This is supported by Y2H assays in which we tested interactions with the N-terminal fragment of TPL (TPL-N), containing the LisH, CTLH and TOP domains which were shown to be essential for binding to the EAR motif and mediate other protein-protein interactions [48], [49]. TIFY8 was unable to bind TPL-N in contrast to NINJA (Figure 3D). This corroborates our previous report showing direct interaction between TPL and NINJA [29], [34]. Next, we performed a yeast-three hybrid assay. In addition to GAL4-AD fused TIFY8 and GAL4DBD fused TPL-N we expressed HisFLAG-NLS fused NINJA in the yeast. Only in presence of NINJA, but not the empty vector, the reporter is activated (Figure 3E). This suggests that that NINJA can act as an adaptor protein between TIFY8 and TPL similar as we have shown for JAZ3 [50].

### TIFY8 acts as a transcriptional repressor

To assess the potential repressor activity of TIFY8 we performed transient expression assays in tobacco protoplasts. TIFY8 was fused to the GAL4DBD and co-expressed with a construct expressing the firefly luciferase (*fLUC*) reporter gene under the control of GAL4 binding elements. Targeting TIFY8 to the UAS promoter reduced basal expression strongly, comparable to the effect of JAZ1:GAL4DBD (Figure 3F). Taken together, our findings demonstrate that TIFY8 acts as a repressor of gene expression, similar to the JAZ proteins [29].

### *TIFY8* expression is repressed by infection with *Pseudomonas syringae*

**Figure 4:**
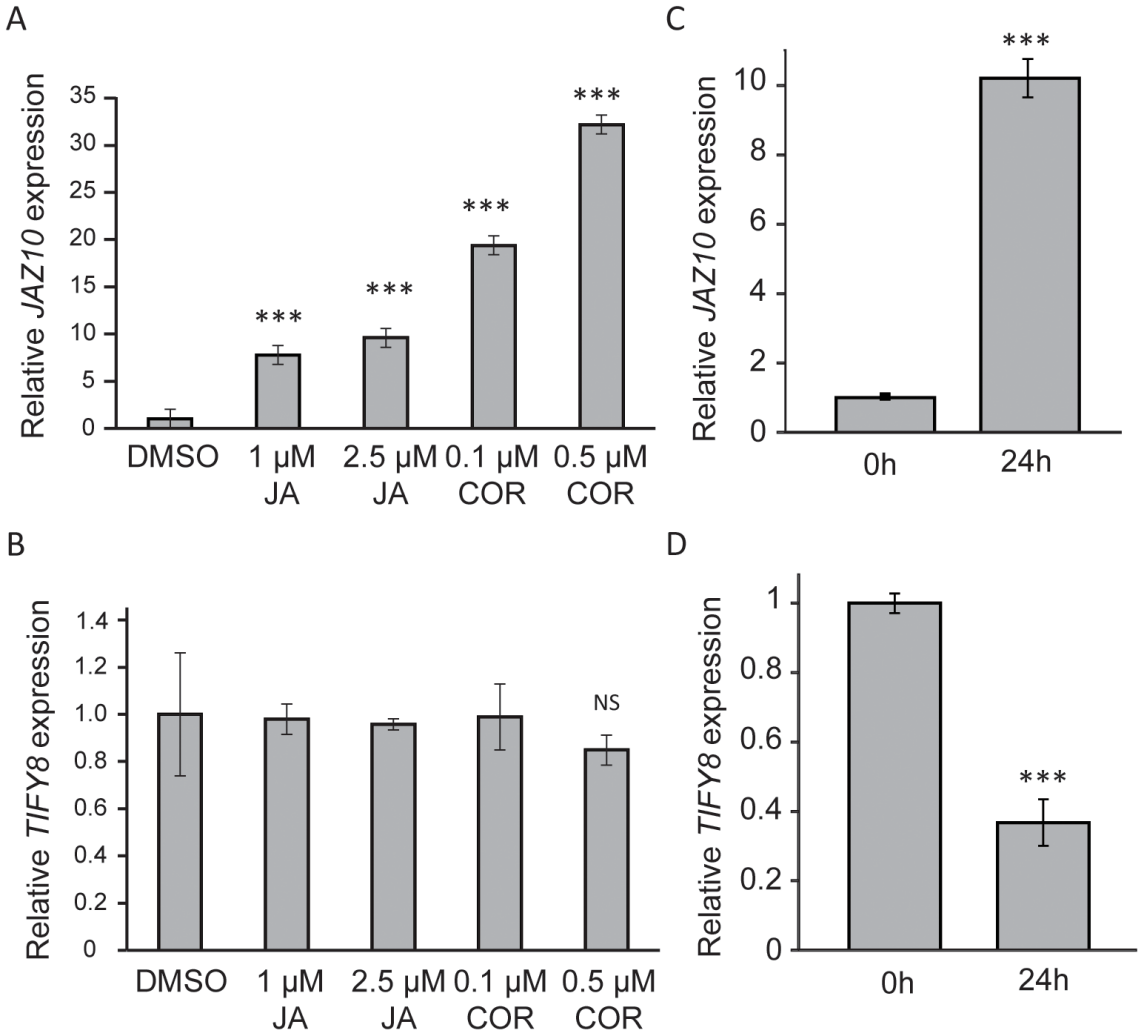
*TIFY8* is not induced by JA and is repressed by *Pst DC3000* infection. **A**–**B**, RT-PCR analysis of *JAZ10* (**A**) *TIFY8* (**B**) expression after JA treatment. Arabidopsis wild-type seeds were germinated on MS media and, after 8 days, transferred to liquid MS media supplied with different concentrations of JA or COR or equivalent amounts of DMSO (mock treatment). Transcript levels were studied 24 h after treatment. Error bars represent ±SE of four biological replicates. **C**–**D**, *JAZ10* and *TIFY8* expression after infection with *Pst* DC3000. Transcript levels were studied in 5-week-old Arabidopsis rosette leaves prior to or 24 h after inoculation with *Pst* DC3000. Error bars represent ±SE of three biological replicates. *UBC* (AT5G25760) was used as internal control and expression values were normalized to those of the wild-type after mock treatment. (NS, p>0.05; ***, p<0.001, t-test).

It is known that *JAZ* transcription is rapidly and highly induced by JAs [14],[15],[29]. Accordingly, *JAZ10* expression in Arabidopsis Col-0 seedlings was induced after 24 h treatment with different concentrations of JA and coronatine (Figure 4A). In contrast, *TIFY8* transcript levels were found not to be altered (Figure 4B). Consulting publicly available microarray data revealed that *TIFY8* gene expression can be altered by different (a)biotic stresses. Amongst them, the hemibiotrophic pathogen *P. syringae* pv. *tomato* DC3000 (*Pst* DC3000) causes transcriptional repression of *TIFY8* (http://www.genevestigator.com; [51]). To validate these observations, bioassays with the virulent *Pst* DC3000 strain were performed. Plant samples of Col-0 wild type plants were harvested prior to and 24 h after inoculation with *Pst* DC3000. *JAZ10* was highly induced (Figure 4C) as reported before [52]. In contrast, *TIFY8* expression was significantly downregulated, in agreement with the public microarray data (Figure 4D).

### Study of TIFY8 expression by promoter::GUS analysis

Analysis of *TIFY8* expression with Genevestigator (http://www.genevestigator.com; [51]) or eFP Browser (http://bar.utoronto.ca/efp/cgi-bin/efpWeb.cgi; [53]) suggested that *TIFY8* might display an expression pattern opposite to that of many of the JAZ members, in particular *JAZ1, JAZ2, JAZ3, JAZ5, JAZ7, JAZ8* and *JAZ10* (Figure S2).

To study the *TIFY8* expression pattern throughout the plant's life span in more detail, we generated transgenic lines carrying a pTIFY8::GUS-GFP reporter construct and compared GUS expression in these lines with that in Arabidopsis lines carrying the pJAZ1::GUS-GFP construct that we previously reported [54].

**Figure 5:**
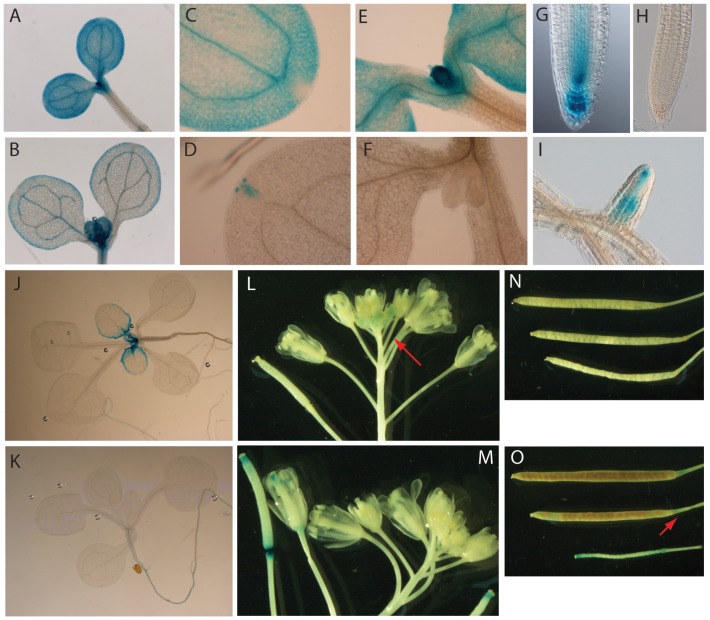
Overview or TIFY8 and JAZ1 promoter gene expression. GUS stains of Arabidopsis plants expressing either *TIFY8* or *JAZ1* promoter fusions to GUS and GFP. **A, B**. *TIFY8* promoter expression pattern in 5 and 7 day-old seedlings, respectively. **C, D**. *TIFY8* (**C**) and *JAZ1* (**D**) promoter expression in 5 day-old cotyledons. **E, F.** *TIFY8* (**E**) and *JAZ1* (**F**) promoter expression in the shoot apical meristem and emerging leaves of 5 day-old seedlings. **G, H**. *TIFY8* (**G**) and *JAZ1* (**H**) promoter expression in the root tip of 5 day-old Arabidopsis seedlings. **I.** *TIFY8* promoter expression in lateral root of a 10 day-old seedling. **J, K.** *TIFY8* (**J**) and *JAZ1* (**K**) promoter expression in 14 day-old seedlings. **L**–**O**. *TIFY8* and *JAZ1* promoter expression in flowers (**L, M**) and siliques (**N, O**).

Sampling seedlings at several time points during early development showed that *TIFY8* is expressed both in shoots and roots. In shoots, the *TIFY8* promoter drives expression in cotyledons and young true leaves, in which expression gradually diminishes towards the base of the leaf during development (Figure 5A, B and J). Strong GUS activity was detected in the shoot apical meristem and emerging leaves (Figure 5E). In roots, we detected very strong activity in the root tip (Figure 5G). In several organs, such as the root tip and cotyledons, pTIFY8-driven expression pattern is opposite to that driven by pJAZ1. In root tips, pTIFY8 is strongly active whereas pJAZ1 seems to be inactive. Conversely, the TIFY8 promoter does not drive reporter gene expression in the cotyledon tip, in contrast to the JAZ1 promoter (Figure 5E-F and 5C–D, respectively). Additionally, pTIFY8-driven GUS activity was found in lateral roots, with high expression levels in the elongating region and the lateral root tip (Figure 5I). In later stages, no GUS expression was detected in mature rosette leaves (data not shown). In flowers, pTIFY8 activity was restricted to younger flowers and not detected in older flowers or siliques, whereas the JAZ1 promoter was active in the stigma at later stages of flower development and in the base and the tip of siliques (Figure 5L–O).

### *TIFY8* overexpression affects primary root growth

**Figure 6:**
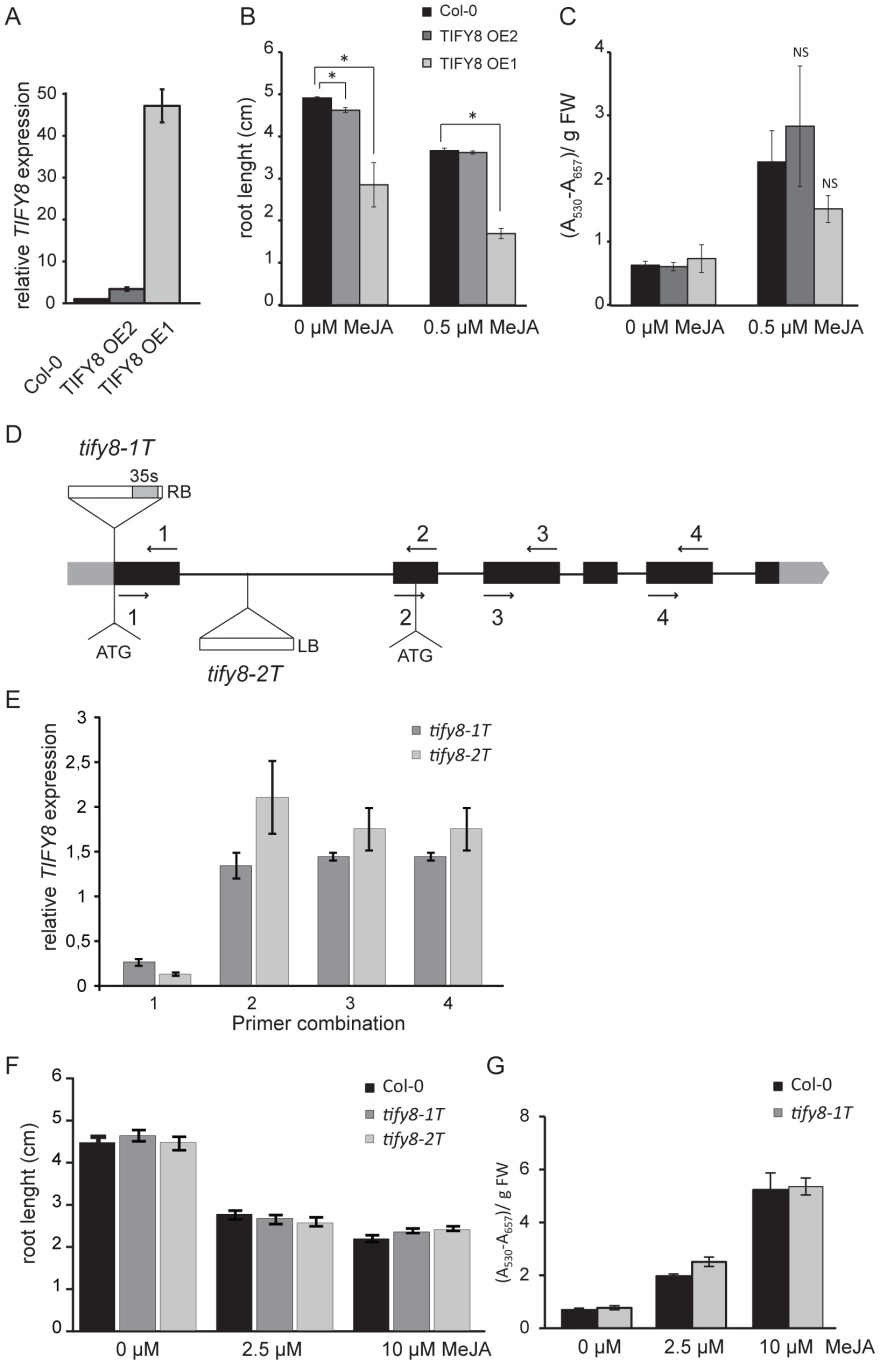
Characterization of transgenic lines with altered *TIFY8* expression. **A**, RT-PCR analysis of *TIFY8* expression levels in two independent TIFY8-OE lines and compared to wild type. Transcript levels were studied in two-week-old Arabidopsis TIFY8-OE and wildtype seedlings. *UBC* (*AT5G25760*) was used as internal control and expression values were normalized to those of the wildtype. Error bars represent ±SE of three technical replicates. **B**–**C**, Analysis of JA-responsiveness of the TIFY8-OE lines compared to wild-type. Root growth inhibition was scored on 11 days after stratification (DAS) (**B**) while anthocyanins were extracted for the same samples used for root growth but harvested 14 DAS (**C**). Four technical repeats per line and treatment, consisting on up to eight seedlings per repeat (20≤n≤32), were analysed. Bars represent average ± SE. Differences between the transgenic lines assayed and wild-type in control conditions are shown (*: p<0.05; t-test). Statistically significant differences for the interaction between genotype and treatment were not found (NS, p>0.05, one-way ANOVA). **D**, Schematic diagram of the *TIFY8* (At4g32570) locus. Black bars, black lines and grey bars represent exons, introns and the untranslated regions, respectively. The T-DNA in the *tify8-1T* line (GK_738B03) is inserted immediately after the start codon of *TIFY8*, and the T-DNA contains the 35S promoter sequence next to the right border (RB). Arrows and numbers indicate different primer combinations covering different regions of *TIFY8*. Primer sequences can be found in Table S2. **E**, RT-PCR analysis of *TIFY8* transcripts in the *tify8-1T* and *tify8-2T* lines. Transcript levels were studied in 1-week-old seedlings. Numbers represent the primer combination used, described in (D). *UBC* (AT5G25760) was used as internal control and expression values (Y-axis) were normalized to those of the wildtype. Error bars represent ±SE of three biological replicates. **F**, Analysis of JA-responsive root growth inhibition of the *tify8-1T* and *tify8-2T* lines compared to wild type performed as in (**B)**. **G**, JA-responsive anthocyanin accumulation in *tify8-1T* performed as in (**C**).

Because of the intriguing expression pattern we decided to generate transgenic lines with altered *TIFY8* expression and assess their phenotypes and response to JAs. First, we generated two independent transgenic lines with enhanced *TIFY8* expression, driven by a pCaMV35S promoter (Figure 6A). We assessed the response to JA by measuring root growth inhibition and anthocyanin accumulation, two of the most commonly used parameters to score JA-responsiveness in Arabidopsis. In the TIFY8-OE lines, root growth (but not anthocyanin accumulation) was already significantly reduced in control conditions in both lines. The TIFY8-OE1 line, which showed strongest TIFY8 overexpression, was also the most affected in root growth (Figure 6A–B), pointing towards an inverse correlation between ectopic *TIFY8* expression levels and root size. Besides this pronounced phenotype, we did not observe significant genotype × treatment effects for root growth inhibition nor anthocyanin accumulation in the TIFY8-OE lines when seedlings were treated with 0.5 µM MeJA (Figure 6B–C). Higher doses than 0.5 µM MeJA rendered plants that were too small and that consequently could not be accurately measured. Overall these data suggest that altered TIFY8 overexpression does not alter the JA response in transgenic plants, at least not under the conditions tested.

### Characterization of TIFY8 T-DNA insertion lines

To further characterize TIFY8 function, we selected a GABI-KAT and a SAIL T-DNA insertion lines [55], [56], referred to as *tify8-1T and tify8-2T*, respectively. Sequencing analysis revealed that the T-DNA was inserted right after the start codon of *TIFY8* in *tify8-1T* and after 219 bp in the first intron in *tify8-2T* (Figure 6D). We investigated the generation of *TIFY8* transcripts by real-time PCR (RT-PCR) in these lines with multiple primer combinations covering the entire length of the gene. Both T-DNA insertions led to aberrant *TIFY8* transcription as a reduction of at least 70% was seen in the levels of transcripts corresponding to the first exon and encoding the N-terminus of TIFY8 (primer combination 1, Figure 6D–E). Unexpectedly, in both lines transcripts of downstream exons were present in levels that slightly exceeded those of wild-type Arabidopsis plants (Figure 6E). The first intron of *TIFY8* is 693 bp long compared to an average and median length of 173 and 101 bp respectively for Arabidopsis [57]. Therefore we cannot exclude that the first intron functions as an alternative promoter of a functional, but truncated, *TIFY8* transcript that misses the first exon and that contains a start codon in frame in exon 2 (Figure 6D). We therefore consider the transcription and function of *TIFY8* similarly disturbed in these two lines, possibly leading to expression, if any, of a truncated TIFY8 protein.

We assessed root growth for both lines and anthocyanin accumulation in *tify8-1T* in absence and presence of JA but no differences were observed compared to Col-0 (Figure 6F–G).

## Discussion

### TIFY8 is not involved in JA signalling

Prompted by the TIFY-family phylogeny (Figure S1) and the interesting developmental expression pattern of *TIFY8*, which is the inverse of many *JAZ* genes (Figure S2), we investigated a possible role of TIFY8 in JA signalling. At the protein level, we could show that the ZIM domain of TIFY8 was functional and facilitated interaction with PPD proteins and NINJA (Figure 2B–C). However, no evidence in Y2H or TAP was obtained for interaction with JAZ proteins although the ZIM domain is known to be responsible for homo- and heterodimerization of JAZ proteins [27], [28]. JA did not influence TIFY8 levels, neither at the transcript or protein level (Figure 2D and Figure 4B). Furthermore, the plant lines with altered *TIFY8* expression that we generated did not show obvious altered JA responses (figure 6B–C and F–G). It is currently unclear in which process TIFY8 is implicated. Overall this suggests that TIFY8 does not directly interfere in JA signalling, though we cannot exclude that it acts in processes that are also regulated by JA signalling, such as root development or resistance to pathogens like *P. syringae*. The advent of new genome editing tools for generating knock-out lines in Arabidopsis holds great promise to study the function of genes such as *TIFY8* with possible complex transcription and insufficient coverage by T-DNA insertion lines [58].

### TIFY8 is a repressor of gene expression

We could show that TIFY8 acts as a transcriptional repressor when targeted to a heterologous promoter (Figure 3F). This corresponds with the proposed recruitment of TPL through the adaptor protein NINJA. Recently, TPL has emerged as the common element of repressor complexes that control a variety of plant processes. At least 219 transcription factors contain the EAR motif responsible for TPL interaction [31]. In addition, transcription factors can recruit TPL via adaptor proteins such as NINJA or the recently described protein TIE1 that links TCP transcription factors to TPL [59]. Accordingly, many TPL interactors have been found in Y2H screens [34], [60], which has led to the coining of the terms “EAR repressorome” and “TPL interactome”.

We could not obtain evidence that TIFY8 binds plant DNA. Therefore, we postulate that it might itself function as an adaptor, binding transcription factors. In agreement with this, the TIFY8 protein has been found to interact with several other proteins in Y2H-screens [34] and TAP analysis (this study). Some of these interactors correspond to transcription or other regulatory factors potentially involved in different pathways. These include INNER NO OUTER (INO, At1g23420, involved in ovule outer integument development, [61]), RESPONSE REGULATOR 14 (ARR14, At2g01760, involved in cytokinin signalling, [62]), ABERRANT LATERAL ROOT FORMATION 14 (ALF4, At5g11030, involved in lateral root development, [63]) from the Y2H-screen and PPD (involved in leaf development, [35]), OBERON (OBE) and TITANIA (TTA) (both involved in embryonic root meristem initiation, [42], [43]), and SPINDLY (involved in cytokinin and gibberellin signalling, [45], [64]) reported here. The *obe1 obe2 and tta1 tta2* double mutants are defective in root development [42], [43]. Therefore, it is tempting to speculate that the expression patterns of *TIFY8* in the root and the root phenotype of the TIFY8-OE1 line correlate with the interaction of TIFY8 with proteins such as OBE2 and TTA2 and postulate a role for TIFY8 in the regulation of root growth and development.

## Experimental Procedures

### Yeast two-hybrid and three-hybrid assays

Yeast two- and three-hybrid analysis was performed as described [65]. In brief, the *Saccharomyces cerevisiae* PJ69-4A yeast strain was co-transformed with bait and prey expressed from pGADT7 en pGBKT7 vectors. Transformants were selected on SD media lacking Leu and Trp (-2). Three individual transformants were grown overnight in liquid (-2) media, and a tenfold dilution of these cultures was dropped on control (-2) and selective media additionally lacking His (-3). Empty vectors were used as negative controls.

In Y3H assays, the MultiSite pMG426 (Ura) vector was used for expression of NINJA, driven by the GDP promoter and C-terminally fused to the SV40 NLS-3xFLAG-6xHis tag ([50]; http://gateway.psb.ugent.be). Yeasts were allowed to grow for 2 days at 30°C before interaction was scored.

### Tandem Affinity Purification (using MALDI TOF/TOF MS)

Entry clones containing the CaMV 35S promoter, the bait ORF and the GS-TAP tag were recombined by MultiSite Gateway LR reaction with pKCTAP as destination vector [66], [67]. Arabidopsis cell suspension cultures (PSB-D) were transformed without callus selection as described previously [68]. Tandem affinity purifications were performed as described [67], [68] with the exception that the soluble protein fraction was obtained by centrifuging twice at 36,900 g for 20 min at 4°C.

Proteolysis and peptide isolation, acquisition of mass spectra by a 4800 MALDI TOF/TOF Proteomics Analyzer (AB SCIEX), and MS-based protein homology identification based on the TAIR genomic database [69] were performed as described in [39]. Experimental background proteins were subtracted based on approximately 40 TAP experiments on wild-type cultures and cultures expressing TAP-tagged mock GUS, RFP and GFP proteins [39].

### Tandem Affinity Purification (using LC-MS/MS analysis)

A downscaled purification protocol was used. In short, cell extracts were made on 2.5 g cell culture and cleared by two subsequent centrifugation steps at 36,900×*g* for 20 minutes. In the first purification step, a protein input of 25 mg was incubated with 25 µl of IgG-Sepharose 6 Fast Flow beads (GE Healthcare). For the second step, 25 µl of Streptavidin Sepharose High Performance (Amersham) was used. Final elution was done with 40 µl 1× NuPAGE sample buffer containing 20 mM Desthiobiotin for 5 minutes. Beads were separated from eluate in a 1-ml Mobicol column (MoBiTec, Göttingen, Germany).

Eluted proteins were separated in a short run of 7 minutes on a 4–12% gradient NuPAGE gel (Invitrogen) and visualized with colloidal Coomassie Brilliant Blue staining. The protein gel was washed for 2 hours in H_2_O, polypeptide disulphide bridges were reduced for 40 min in 25 mL of 6,66 mM DTT in 50 mM NH_4_HCO_3_ and sequentially the thiol groups were alkylated for 30 min in 25 mL 55 mM IAM in 50 mM NH_4_HCO_3_. After washing with H_2_O, a broad zone containing the proteins was cut from the protein gel, sliced into 24 gel plugs, and collected together in a single Eppendorf. Gel plugs were washed twice with H_2_O, dehydrated with 95% CH_3_CN (v/v), rehydrated with H_2_O and dehydrated again with 95% CH_3_CN (v/v). Dehydrated gel particles were rehydrated in 60 µL digest buffer containing 750 ng trypsin (MS Gold; Promega, Madison, WI), 50 mM NH_4_HCO_3_ and 10% CH_3_CN (v/v) for 30 min at 4°C. Proteins were digested at 37°C for 3.5 hours.

The obtained peptide mixtures were introduced into an LC-MS/MS system, the Ultimate 3000 RSLC nano (Dionex, Amsterdam, The Netherlands) in-line connected to an LTQ Orbitrap Velos (Thermo Fisher Scientific, Bremen, Germany). The sample mixture was loaded on a trapping column (made in-house, 100 µm internal diameter (I.D.) ×20 mm (length), 5 µm C18 Reprosil-HD beads, Dr. Maisch GmbH, Ammerbuch-Entringen, Germany). After back-flushing from the trapping column, the sample was loaded on a reverse-phase column (made in-house, 75 µm I.D. ×150 mm, 5 µm C18 Reprosil-HD beads, Dr. Maisch). Peptides were loaded with solvent A (0.1% trifluoroacetic acid, 2% acetonitrile), and separated with a linear gradient from 2% solvent A' (0.1% formic acid) to 50% solvent B' (0.1% formic acid and 80% acetonitrile) at a flow rate of 300 nL/min, followed by a wash step reaching 100% solvent B'.

The mass spectrometer was operated in data-dependent mode, automatically switching between MS and MS/MS acquisition for the ten most abundant peaks in a given MS spectrum. In the LTQ Orbitrap Velos, full scan MS spectra were acquired in the Orbitrap at a target value of 1E6 with a resolution of 60,000. The ten most intense ions were then isolated for fragmentation in the linear ion trap, with a dynamic exclusion of 20 seconds. Peptides were fragmented after filling the ion trap at a target value of 1E4 ion counts.

From the MS/MS data in each LC run, Mascot Generic Files were created using the Mascot Distiller software (version 2.4.1.0, Matrix Science, www.matrixscience.com/Distiller.html). When generating these peak lists, grouping of spectra was allowed with a maximum intermediate retention time of 30 seconds and a maximum intermediate scan count of 5 was used where possible. Grouping was done with 0.005 Da precursor tolerance. A peak list was only generated when the MS/MS spectrum contained more than 10 peaks. There was no de-isotoping and the relative signal-to-noise limit was set to 2. These peak lists were then searched with the Mascot search engine (version 2.3, MatrixScience, www.matrixscience.com) using the Mascot Daemon interface (Matrix Science, www.matrixscience.com). Spectra were searched against the TAIR10 database containing 35386 sequence entries. Variable modifications were set to methionine oxidation and methylation of aspartic acid and glutamic acid. Fixed modifications were set to carbamidomethylation of cysteines. Mass tolerance on MS was set to 10 ppm (with Mascot's C13 option set to 1) and the MS/MS tolerance at 0.5 Da. The peptide charge was set to 1+, 2+ and 3+ and the instrument setting was set to ESI-TRAP. Trypsin was set as the protease used, allowing for 1 missed cleavage, and also cleavage was allowed when arginine or lysine is followed by proline. Only high confident peptides, ranked one and with scores above the threshold score, set at 99% confidence, were withheld. Only proteins with at least two matched high confident peptides were retained.

A list of non-specific background proteins was assembled by combining our previous background list [39] with background proteins from control GS purifications on mock, GFP-GS, and GUS-GS cell culture extracts identified with LTQ Orbitrap Velos. To obtain the final list of interactors, these background proteins were subtracted from the list of identified proteins.

### Transient expression assays

Transient expression assays were performed as described previously [70]. Protoplasts were prepared from a Bright Yellow-2 (BY-2) tobacco cell culture and co-transfected with a reporter plasmid containing the firefly-Luciferase (fLUC) reporter gene driven by a promoter containing five GAL4-binding sites, a normalization construct expressing Renilla luciferase (rLUC) under the control of the 35S promoter and effector constructs. GAL4DBD fusions were generated by combining pEN-L4-2-R1 (35S promoter), pEN-R2-GAL4DBD-L3 and an entry clone holding the ORF, combined by MultiSite Gateway LR reaction with pm43GW7 as destination vector. For each experiment, 2 µg of each plasmid were used. After transfection, protoplasts were incubated overnight in the dark, at room temperature and with gentle agitation. The next day, protoplasts were lysed, and fLUC and rLUC activities were determined with the Dual-Luciferase reporter assay system (Promega). Variations in transfection efficiency and technical error were corrected by normalization of fLUC by rLUC activities. All transactivation assays were conducted in an automated experimental set-up. A one-way ANOVA and Tukey HSD's Post Hoc test were performed to confirm statistically significant differences between control and effector constructs (p<0.05).

### Generation of plant lines

The T-DNA knock-out lines *tify8-1T* and *tify8-2T* were retrieved from GABI-KAT and SAIL respectively [55], [56] and genotyped by PCR as homozygous for the T-DNA insertion in the Col-0 ecotype background.For generation of transgenic plants with 35S promoter-driven *TIFY8* overexpression fused C-terminally to GFP (TIFY8-GFP) or to a TAP tag (TIFY8-GS) a destination clone containing the full-length *TIFY8* was retrieved from ABRC (DKLAT4G32570). From this construct, an entry clone without stop codon was generated by reverse BP reaction into the entry vector pDONR221 and used for recombination with the pK7FWG2 and the pKCTAP destination vectors, respectively [68], [71] using the Gateway LR II kit (Invitrogen), yielding the corresponding expression clones. For overexpression without a fusion tag (TIFY8-OE), the ABRC entry clone G22977 was recombined with the pFAST-G02 vector [72].For gene promoter expression assays, a 1175 bp fragment of the TIFY8 promoter was retrieved from the Arabidopsis Promoterome database (www.psb.ugent.be/SAP) and amplified by PCR to be cloned in the pDONRP4P1R entry vector. The entry clone was then used for LR reaction with the pmK7S*NFm14GW destination vector [73], yielding the *TIFY8promoter*::GUS-GFP expression clone.Following cloning and sequence verification, the expression vectors were transformed into *Agrobacterium tumefaciens* C58C1 (pMP90) by electroporation. Transgenic Arabidopsis seeds were generated by floral dip, using Col-0 as the background ecotype. Transformants were selected on MS media supplied with the corresponding antibiotic and homozygous T3 plant lines were used in the assays.

### *In vitro* plant growth conditions

For all the experiments using plants grown *in vitro* described, Arabidopsis seedlings were sterilized by the chlorine gas method and shown on sterile plates containing the corresponding growth media. Plates were kept in the dark at 4°C and 2days for stratification. Then, plates were transferred to a growth room with 21°C temperature and a 16 h light/8 h dark light regime. The day of the transfer was considered as 0 days after stratification (0 DAS).

### Confocal microscopy

Plants expressing the 35S::TIFY8-GFP fusion, 35S::GFP and 35S::NLS-GFP were germinated on solid MS plates (containing 10 g/L of sucrose and 8 g/L agar) placed vertically. On the day of imaging, seedlings were briefly incubated in propidium iodide (3 mg/L, Sigma) and subsequently washed and mounted in milliQ water. Fluorescence microscopy was performed with an Olympus FV10 ASW confocal microscope.

### Protein degradation assays

For the study of TIFY8 stability, homozygous transgenic lines producing either TIFY8 or JAZ1 fused to a protein G-SBP (GS) C-terminal tag (TIFY8- or JAZ1-GS) were used. Seeds were grown in liquid MS media with 10 g/L sucrose (pH 5.7) for 7 days. Seedlings were treated with 50 µM JA or ethanol (mock) for one hour. Total protein was extracted and 20 µg loaded on a 4–15% TGX gel (Bio-Rad,) and run for 20 min at 300 V. Next, blotting was performed with Trans-blot Turbo transfer 0.2 µm PVDF membranes (Bio-Rad). A 1/2500 dilution of Peroxidase Anti-Peroxidase (PAP) antibody (P1291, Sigma-Aldrich) was used for protein G detection. For detection of CDKA;1, anti-cdc2 PSTAIR (sc-53, Santa Cruz) was used. Chemiluminescent detection was performed with Western Bright ECL (Isogen, http://www.isogen-lifescience.com/).

### JA and COR treatment of Arabidopsis wild-type seedlings

Wild-type seedlings were grown as described and, 8 DAS, transferred to liquid MS media containing the corresponding final JA or COR concentration, whereas DMSO was used as control treatment. After 24 hours, the seedlings were harvested and frozen on liquid Nitrogen.

### Semiquantitative RT-PCR analysis

Frozen plant material was ground in a Retsch MM300 mixer and total RNA was extracted using the Qiagen RNeasy kit (Qiagen, http://www.qiagen.com/). An RNase-free DNase step was performed following manufacturer's instructions for preparation of RNA. Next, 1 µg of total RNA was used for cDNA synthesis with the iScript kit (Bio-Rad, http://www.bio-rad.com/). RT-PCR was performed on a LightCycler 480 system (Roche, http://www.roche.com) using the Fast Start SYBR Green I PCR mix (Roche). The primer sequences are provided as Table S2. For *TIFY8* primer pair #4 was used unless stated otherwise. Samples were amplified as described: one pre-incubation step (95°C, 10 s) followed by 45 amplification cycles (incubation 95°C for 10 s, annealing at 65°C for 15 s, elongation at 72°C for 15 s). Primer efficiency was at least 1.7. Gene expression levels were quantified relative to the housekeeping gene *UBC* (*AT5G25760*).

### β-Glucuronidase (GUS) stains

Samples were harvested on Falcon multiwell plates and kept in 90% acetone to clear the tissue. Next, the acetone was removed and replaced by the GUS staining solution containing 2 mM X-Gluc solution in N,N-dimethylformamide (DMF), 0.1 M NaPO_4_ pH 7.0, 1 mM K_3_Fe(CN)_6_ and 0.1% Triton X-100 diluted in distilled water. The samples were incubated at 37°C until blue coloration appeared. Next, the GUS staining solution was removed and the plant tissue was cleared with 70% ethanol at 4°C overnight. Sample imaging was performed either in a light microscope (Leica BXL51) or a binocular (Leica MZ16).

### Root growth assay

Seedlings were sown on MS media plates provided with 10 g/L sucrose, 8 g/L agar, pH 5,7 and the corresponding final MeJA concentration. Following stratification, plates were placed vertically under the conditions described. Plates were scanned at 11 DAS at a 300 dpi resolution and root length was measured by means of the EzRhizo software (http://www.root-image-analysis.org/ez-rhizo). Samples were kept in the growth room for another three days for anthocyanin accumulation measurements.

### Anthocyanin accumulation

At 14 DAS, samples from the root growth assay were harvested and weighted in pre-frozen 1.5 mL Eppendorf tubes provided with 3-mm metal balls. Samples were frozen on liquid nitrogen and ground in a Retsch MM300 mixer. For anthocyanin extraction, each sample was added 750 µL of extraction buffer (MeOH HCl 1%) and kept rotating in the dark for 10 minutes. Next, 500 µL of water and 200 µL of chloroform were added, mixing inverting the tubes after each step. Samples were centrifuged for 5 min at full speed and 200 µL of the supernatant were transferred to a 96-well plate. Anthocyanin accumulation was measured as A_530_-A_657_ and referred to mg of fresh weight.

### Infection of Arabidopsis plants with *P. syringae* pv. *tomato* DC3000

The virulent pathogen *P. syringae* pv. *tomato* (*Pst*) DC3000 was incubated overnight at 28°C in liquid King's B (KB) medium as described previously [74]. Bacterial cells were collected by centrifugation (10 min, 2000×*g*) and resuspended in 10 mM MgSO_4_ to a final density of 5×10^6^ colony-forming units per ml (CFU/ml). This suspension was used for pressure-infiltration of leaves of 5-week-old Arabidopsis plants as described [74]. Leaves were harvested prior to and 24 h after inoculation with *Pst* DC3000. Three biological replicates per genotype and time point were analysed.

## Accession Numbers

Arabidopsis Genome Initiative (AGI) accession numbers for the genes studied: TIFY8 (At4g32570), NINJA (At4g28910), TPL (At1g15750), PPD2 (At4g14720) and JAZ1 (At1g19180). AGI codes of interacting proteins identified by tandem affinity purification are listed in Table 1.

## Supporting Information

Figure S1 AtTIFY8 orthologues in other plant species. Blue and red colours represent the existence or absence of putative AtTIFY8 orthologues in different species covered by the PLAZA comparative genomics resource. Numbers in brackets indicate the number of putative orthologues in each species (http://bioinformatics.psb.ugent.be/plaza). Orthologous gene families were inferred through sequence-based clustering with OrthoMCL [38].
(TIF)

Figure S2 The *TIFY8* expression pattern is opposite to that of *JAZ*. A. Schematic representation of *TIFY8* and *JAZ* gene expression patterns in different plant tissues, based on the hierarchical clustering of publicly available microarray data (www.genevestigator.com, [51]). *JAZ4* and *JAZ11* were not included since microarray data are not available. *JAZ12* was not studied as it is highly expressed in all tissues. B, C. Expression patterns of *TIFY8* (B) and *JAZ10* (C) extracted from the eFP Browser. (http://www.bar.utoronto.ca/efp; [53]).
(TIF)

Table S1 MALDI-TOF/TOF-MS identification of TIFY8 interactors.
(PDF)

Table S2 Primers used in this study.
(PDF)

Dataset S1 Protein Identification details obtained with the LTQ Orbitrap.
(XLSX)
