# Supplementary figures and images for: The Non-JAZ TIFY Protein TIFY8 from Arabidopsis thaliana Is a Transcriptional Repressor
Source: PLoS One. 2014 Jan 8;9(1):e84891. doi: 10.1371/journal.pone.0084891 (PMC3885651; doi:10.1371/journal.pone.0084891)

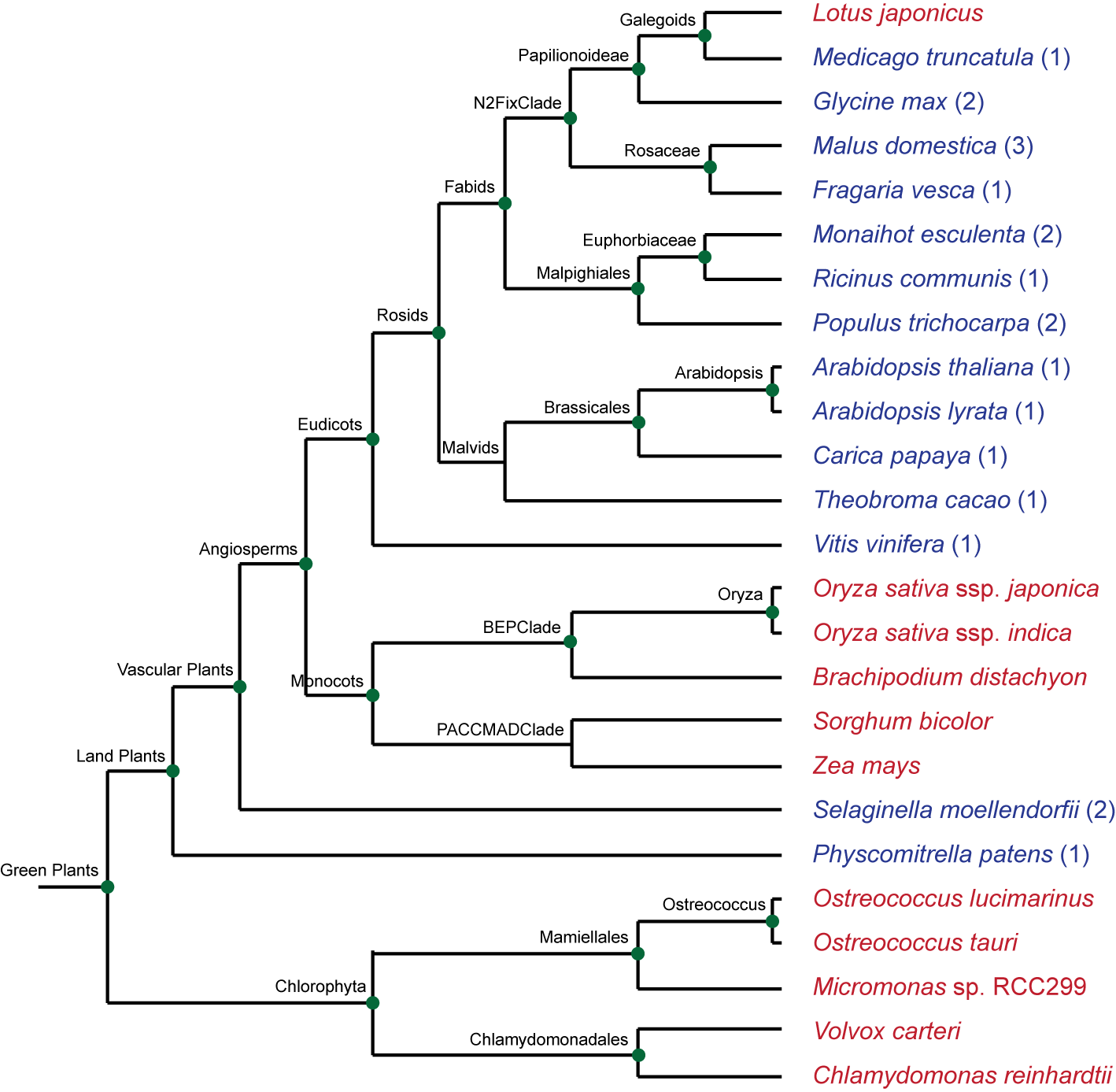

Supplement: Figure S1 — AtTIFY8 orthologues in other plant species. Blue and red colours represent the existence or absence of putative AtTIFY8 orthologues in different species covered by the PLAZA comparative genomics resource. Numbers in brackets indicate the number of putative orthologues in each species (http://bioinformatics.psb.ugent.be/plaza). Orthologous gene families were inferred through sequence-based clustering with OrthoMCL [38]. (TIF) [file pone.0084891.s001.tif]

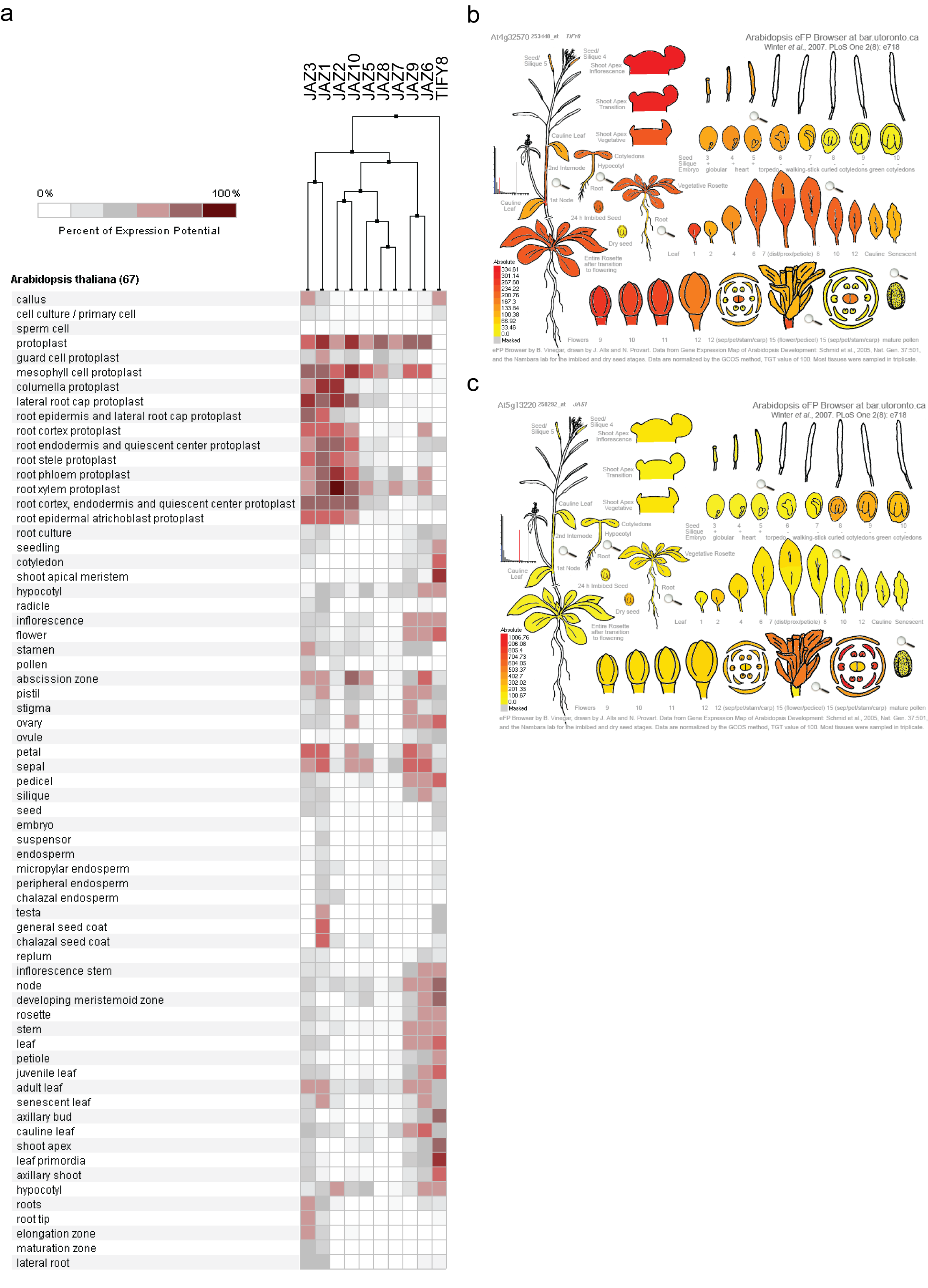

Supplement: Figure S2 — The TIFY8 expression pattern is opposite to that of JAZ. A. Schematic representation of TIFY8 and JAZ gene expression patterns in different plant tissues, based on the hierarchical clustering of publicly available microarray data (www.genevestigator.com, [51]). JAZ4 and JAZ11 were not included since microarray data are not available. JAZ12 was not studied as it is highly expressed in all tissues. B, C. Expression patterns of TIFY8 (B) and JAZ10 (C) extracted from the eFP Browser. (http://www.bar.utoronto.ca/efp; [53]). (TIF) [file pone.0084891.s002.tif]
