# Supplementary material for: The Non-JAZ TIFY Protein TIFY8 from Arabidopsis thaliana Is a Transcriptional Repressor
Source: PLoS One. 2014 Jan 8;9(1):e84891. doi: 10.1371/journal.pone.0084891 (PMC3885651; doi:10.1371/journal.pone.0084891)
Supplement: Table S1 — MALDI-TOF/TOF-MS identification of TIFY8 interactors. (PDF) [file pone.0084891.s003.pdf]

Supplementary Table S1. MALDI-TOF/TOF-MS identification of TIFY8 interactors.

Prey			PMF data					MSMS data													
Prey Locus	Prey Name	Found/ 2 exp[1]	Protein Score	E-value[2]	RMS error (ppm)	Sequence coverage %	Unique Peptides[3]	Total Ion Score	Number[4]	Start	End	Observed	Mr(Exp)	Mr(Calc)	Delta	Miss	Ion Score	Expect	Peptide	Modification
AT4G14720	PPD2	2	217	7,10E-18	8	31	9	162	*1*	*76*	*86*	1217,6917	1216,684	1216,6928	-0,0084	0	25	5,90E-02	ILVSQPPNPPR	
									2	18	29	1417,7452	1416,738	1416,746	-0,0081	0	61	2,40E-05	LLTEEDISQLTR	
									3	60	74	1533,7501	1532,743	1532,7471	-0,0042	0	48	3,80E-04	ALYEPGDDSGAGILR	
									4	179	200	2403,1855	2402,178	2402,1689	0,0094	0	28	3,00E-02	SIMHFAANPIDLPENGIFASSR	Oxidation (M)
AT4G28910	NINJA	2	73	1,70E-03	4	13	3	60	1	276	292	1618,8217	1617,814	1617,8111	0,0033	0	60	2,20E-05	DGSGGIVALSQSPFAGR	
AT4G32295	unknown	2						36	1	26	34	1088,6483	1087,641	1087,639	0,0021	0	36	2,40E-03	GLLIQEIFR	

[1]Number of times that a prey was found in 2 independent TAP experiments with the respective bait
[2]The E-value in Mascot corresponds to the number of times one would expect this score by chance alone
[3]Unique peptides found per identification. Peptides are not present in control TAP experiments with eg. GFP or GUS
[4]Number of peptides from which a sequence was obtained
