# Supplementary material for: The Non-JAZ TIFY Protein TIFY8 from Arabidopsis thaliana Is a Transcriptional Repressor
Source: PLoS One. 2014 Jan 8;9(1):e84891. doi: 10.1371/journal.pone.0084891 (PMC3885651; doi:10.1371/journal.pone.0084891)
Supplement: Table S2 — Primers used in this study. (PDF) [file pone.0084891.s004.pdf]

Supplementary Table S2. Primers used in this study

ID		Sequence	Use
TIFY8-N	Fw	GGGGACAAGTTTGTACAAAAAAGCAGGCTTA**ATG**ATGGTGAACCACAAC	Figure 2B-C
TIFY8-N	Rv	GGGGACCACTTTGTACAAGAAAGCTGGGTA**TCA**CCGATTTCCGGTATTTGAAGGG	Figure 2B-C
TIFY8-C	Fw	GGGGACAAGTTTGTACAAAAAAGCAGGCTTA**ATG**AAAGACTTGGCGTCG	Figure 2B-C
TIFY8-C	Rv	GGGGACCACTTTGTACAAGAAAGCTGGGTA**TCA**TGTGGCTTCTTTTTC	Figure 2B-C
TPL-N	Fw	GGGGACAAGTTTGTACAAAAAAGCAGGCTCC**ATG**TCTTCTCTTAGTAGAGAG	Figure 3D
TPL-N	Rv	GGGGACCACTTTGTACAAGAAAGCTGGGTC**TCM**ATTTTTACAAAGCTGGTGTTG	Figure 3D
JAZ10	Fw	ACGCTCCTAAGCCTAAGTTCC	Figure 4A
JAZ10	Rv	TCGAAATCGCACCTTGAATA	Figure 4A
UBC	Fw	CTGCGACTCAGGGAATCTTCTAA	Figure 4+6
UBC	Rv	TTGTGCCATTGAATTGAACCC	Figure 4+6
TIFY8 prom	Fw	GGGGACAACTTTGTATAGAAAAGTTGTCCTGAAGAGCCTCGATTTT	Figure 5
TIFY8 prom	Rv	GGGGACTGCTTTTTTGTACAAACTTGGAAACCAGTGTGTCAGTGTGG	Figure 5
TIFY8-1	Fw	CCGACAGACAGAACAAGATAAGC	Figure 6E
TIFY8-1	Rv	AAGCAGAAGCCGTGGAAGG	Figure 6E
TIFY8-2	Fw	GGGAATCATCTTGATGGGATAC	Figure 6E
TIFY8-2	Rv	CCTGAAAACCGATTGCTCAT	Figure 6E
TIFY8-3	Fw	CCATCAGTTATTGCTCAGACAG	Figure 6E
TIFY8-3	Rv	GCTTGAGTTTGGCATTGTAAAAG	Figure 6E
TIFY8-4	Fw	AGGCGGATGTGATAATGG	Figure 6E
TIFY8-4	Rv	CTGGAGGTAGGTTGATGAC	Figure 6E

TCM: reverse primers were ambiguity coded to obtain both clones with stop codon (TGA) and without (GGA).
